# Supplementary material for: Dereplication by High-Performance Liquid Chromatography (HPLC) with Quadrupole-Time-of-Flight Mass Spectroscopy (qTOF-MS) and Antiviral Activities of Phlorotannins from Ecklonia cava
Source: Mar Drugs. 2019 Mar 4;17(3):149. doi: 10.3390/md17030149 (PMC6471242; doi:10.3390/md17030149)
Supplement: Supplementary file 1 [file marinedrugs-17-00149-s001.pdf]

Supplementary Materials:

Dereplication by High-Performance Liquid Chromatography (HPLC) with Quadrupole-Time-of-Flight Mass Spectroscopy (qTOF-MS) and Antiviral Activities of Phlorotannins from *Ecklonia cava*

Hyo Moon Cho [1,†], Thi Phuong Doan [1,†], Thi Kim Quy Ha [1], Hyun Woo Kim [1], Ba Wool Lee [1], Ha Thanh Tung Pham [1], Tae Oh Cho [2] and Won Keun Oh [1,*]

[1] Korea Bioactive Natural Material Bank, Research Institute of Pharmaceutical Sciences, College of Pharmacy, Seoul National University, Seoul 08826, Korea; chgyans@naver.com (H.M.C.); phuongdoan@snu.ac.kr (T.P.D.); htkquy@ctu.edu.vn (T.K.Q.H.); kimkami2@snu.ac.kr (H.W.K.); paul36@snu.ac.kr (B.W.L.); thtungdl@gmail.com (H.T.T.P.)

[2] Marine Bio Research Center, Department of Life Science, Chosun University, Gwangju 501-759, Korea; tocho@chosun.ac.kr

* Correspondence: wkoh1@snu.ac.kr; Tel.: +82-02-880-7872

† These authors contributed equally.

Contents

Figure S1. HRESIMS spectrum of compound **1**............... **Error! Bookmark not defined.**

Figure S2. IR spectrum of compound **1**. 5

Figure S3. ^{1}H NMR spectrum of compound **1** (800 MHz, DMSO-d_6)............................... 6

Figure S4. ^{13}C NMR spectrum of compound **1** (200 MHz, DMSO-d_6)**Error! Bookmark not defined.**

Figure S5. HSQC spectrum of compound **1** (800 MHz, DMSO-d_6)**Error! Bookmark not defined.**

Figure S6. HMBC spectrum of compound **1** (800 MHz, DMSO-d_6) 10

Figure S7. ROESY spectrum of compound **1** (800 MHz, DMSO-d_6)**Error! Bookmark not defined.**

Figure S8. HRESIMS spectrum of compound **2**.. 11

Figure S9. IR spectrum of compound **2**. 12

Figure S10. ^{1}H NMR spectrum of compound **2** (850 MHz, DMSO-d_6)........................... 13

Figure S11. ^{13}C NMR spectrum of compound **2** (212.5 MHz, DMSO-d_6)....................... 14

Figure S12. HSQC spectrum of compound **2** (850 MHz, DMSO-d_6) 15

Figure S13. HMBC spectrum of compound **2** (850 MHz, DMSO-d_6) 16

Figure S14. ROESY spectrum of compound **2** (850 MHz, DMSO-d_6)............................ 17

Figure S15. Relationships between ion masses (*m/z* value) in negative ion mode and RMD values for compounds detected by HPLC-qTOFMS in the EC70 fraction 18

Figure S16. HPLC-qTOFMS measurement for twelve isolated compounds in negative ion mode at collision energy of 50 eV 19

Figure S17. MS/MS spectra and fragment ions analysis of isolated compounds. HPLC-qTOFMS spectroscopic data of these single compounds were measured in negative mode at collision energy of 50 eV……………………………………………………………20

Figure S18. Effects of compound **11**, **12**, **13**, and **14** on the viral protein synthesis at a concentration of 20 µM; original uncropped blots 21

Figure S19. Inhibitory effects of compound **12** on the viral proteins synthesis in a concentration-dependent manner; original uncropped blots.......................................22-23

Figure S20. (A) Key HMBC correlations of compounds **1** and **2**. (B) Key ROESY correlations on 3D structure of compounds **1** and **2**………………………………………24

Figure S21. Physicochemical properties of isolated known compounds from *E. cava*.25-28

Table S1. List of species in *Ecklonia* genus from website World Register of Marine Species (http://www.marinespecies.org).. 29

Table S2. The inhibitory effects of compounds **11**, **12**, **13**, and **14** against the H1N1 A/PR/8/34 virus in a cytopathic effect and cytotoxicity assays.. 30

Table S3. ^{1}H and ^{13}C NMR spectroscopic data of compounds **1** and **2** in DMSO-d_6 (δ in ppm) ……………………………………………………………………………...31

Scheme S1. Extraction and fractionation procedure of *E. cava*…………………………..32

Figure S1. HRESIMS spectrum of compound **1.**

Qualitative Compound Report

**Data File**	EC2-3-2_1.d	**Sample Name**	EC2-3-2
**Sample Type**	Sample	**Position**	p1f2
**Instrument Name**	Instrument 1	**User Name**	6530-PC\admin
**Acq Method**	DIP method-0p.m	**Acquired Time**	11/8/2018 10:02:21 AM
**IRM Calibration Status**	Success	**DA Method**	Default.m
**Comment**			

Sample Group **Info.**

Stream Name LC 1

Compound Table

Compound Label	RT	Mass	Abund	Formula	Tgt Mass	Diff (ppm)
Cpd 1: C36H24O18	0.207	744.0968	106771	C36H24O18	744.0963	0.69

Compound Label	RT	Algorithm	Mass
Cpd 1: C36H24O18	0.207	Find By Formula	744.0968

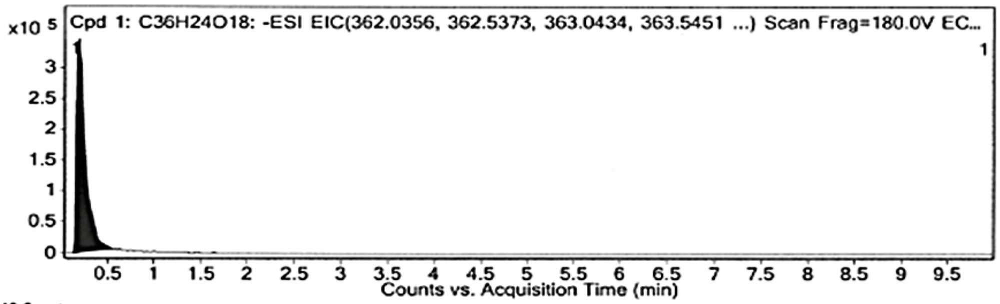

MS Spectrum

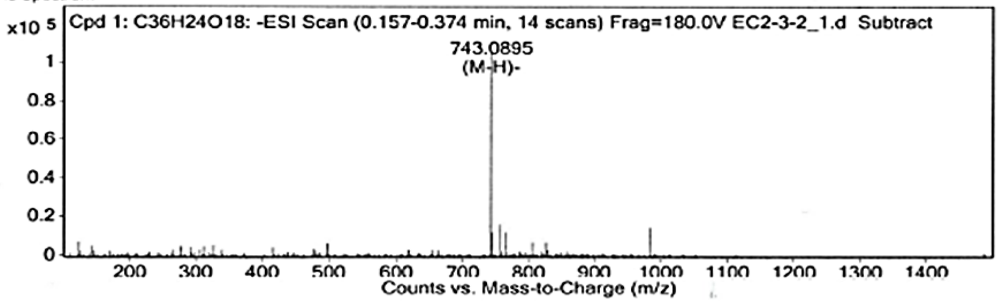

MS Spectrum Peak List

m/z	*Calc m/z*	Diff(ppm)	z	Abund	Formula	Ion
725.0766	725.0784	-2.54	-1	289	C36 H21 O17	(M-H)-[-H2O]
743.0895	743.089	0.69		106771	C36 H23 O18	(M-H)-
744.093	744.0924	0.8		41729	C36 H23 O18	(M-H)-
745.0952	745.0949	0.33		12163	C36 H23 O18	(M-H)-
746.0982	746.0976	0.71		2990	C36 H23 O18	(M-H)-
761.079	761.0551	31.45	-1	326	C36 H22 Cl O17	(M+Cl)-[-H2O]
771.0737	771.0839	-13.23	-1	277	C37 H23 O19	(M+HCOO)-[-H2O]
779.0483	779.0657	-22.35	-1	1008	C36 H24 Cl O18	(M+Cl)-
789.0926	789.0945	-2.36	-1	2191	C37 H25 O20	(M+HCOO)-
1487.1826	1487.1853	-1.8	-1	892	C72 H47 O36	(2M-H)-

--- End Of Report ---

Figure S2. IR spectrum of compound **1.**

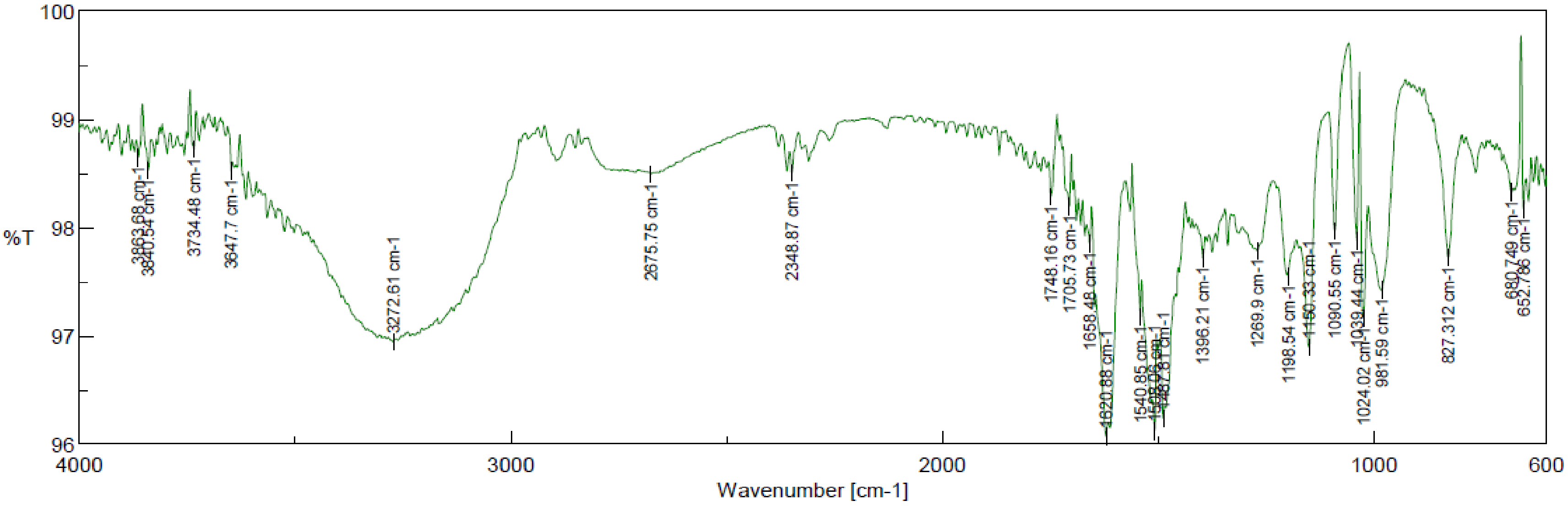

[Comment]
Sample Name
Comment
User
Division
Company 공동기기실

[Data Information]
Creation Date 2018-10-26 오전 11:20
Data array type Linear data array
Horizontal Wavenumber [cm-1]
Vertical %T
Start 599.753 cm-1
End 4000.6 cm-1
Data pitch 0.964233 cm-1
Data points 3528

[Measurement Information]
Model Name FT/IR-4200typeA
Serial Number B038361018

Light Source Standard
Detector TGS
Accumulation 16
Resolution 4 cm-1
Zero Filling On
Apodization Cosine
Gain Auto (2)
Aperture Auto (7.1 mm)
Scanning Speed Auto (2 mm/sec)
Filter Auto (30000 Hz)

EC2-3.2

Figure S3. ^{1}H NMR spectrum of compound **1** (800 MHz, DMSO-d_6).

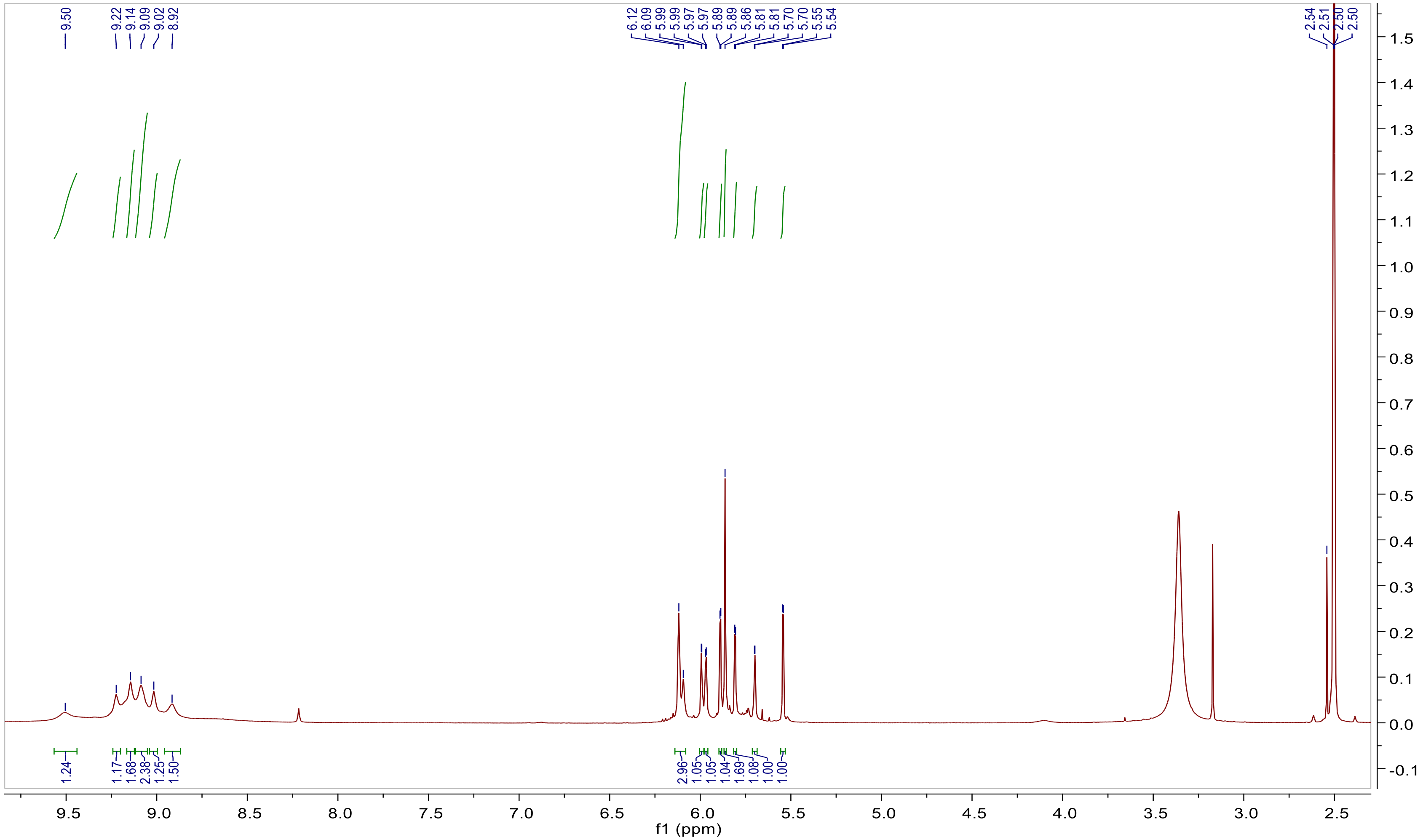

Figure S4. ^{13}C NMR spectrum of compound **1** (200 MHz, DMSO-d_6).

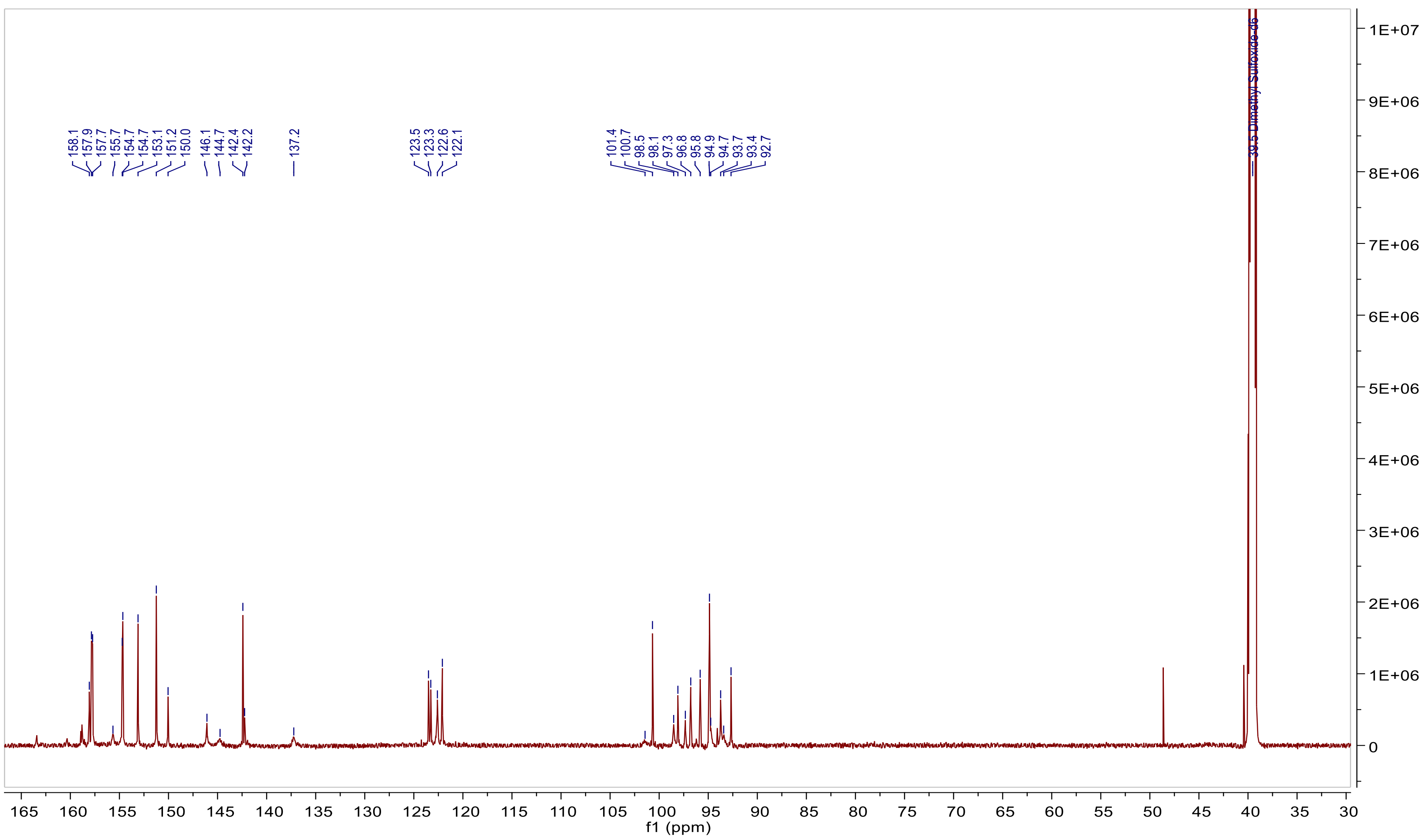

Figure S5. HSQC spectrum of compound **1** (800 MHz, DMSO-d_6).

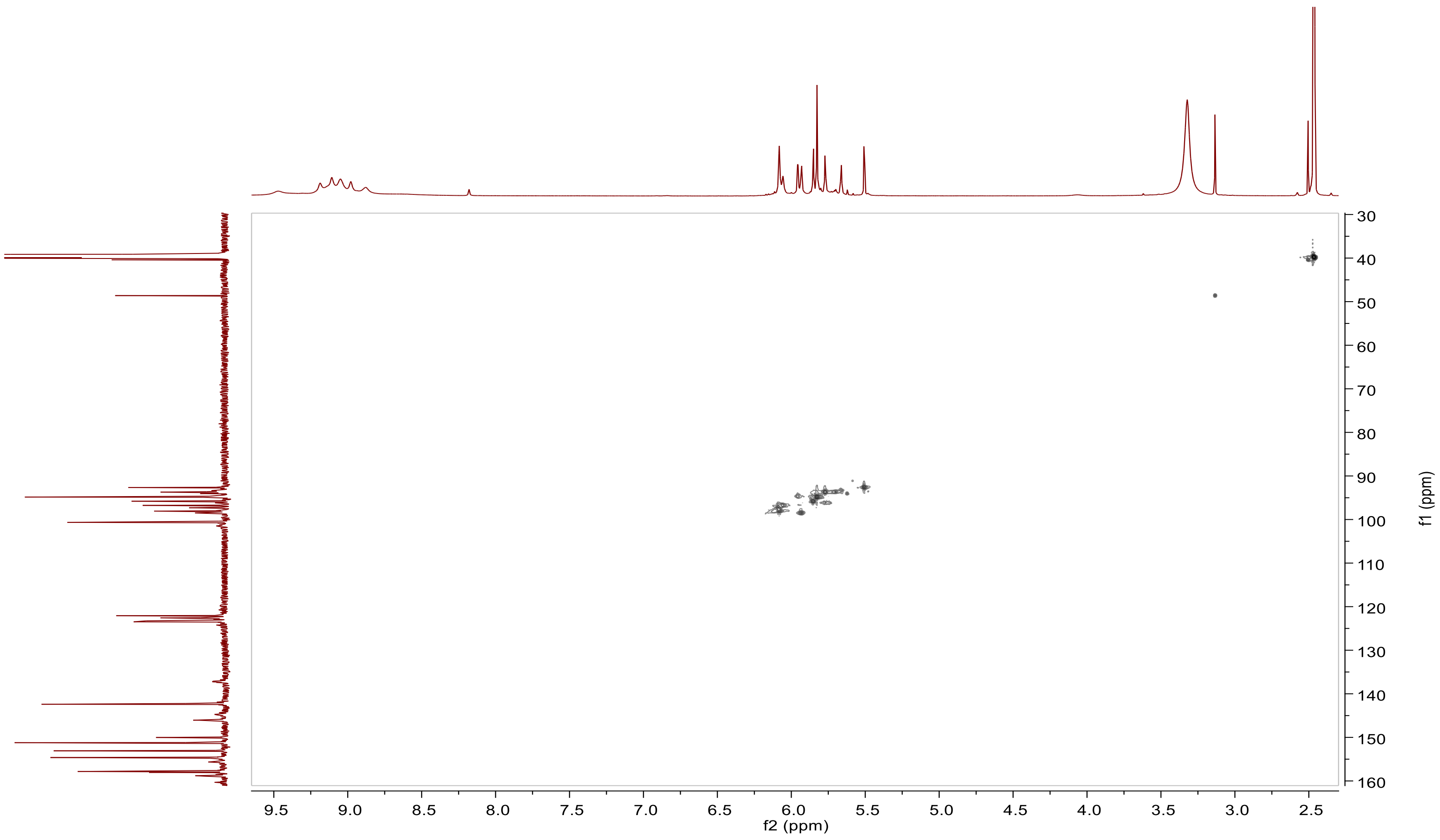

Figure S6. HMBC spectrum of compound **1** (800 MHz, DMSO-d_6).

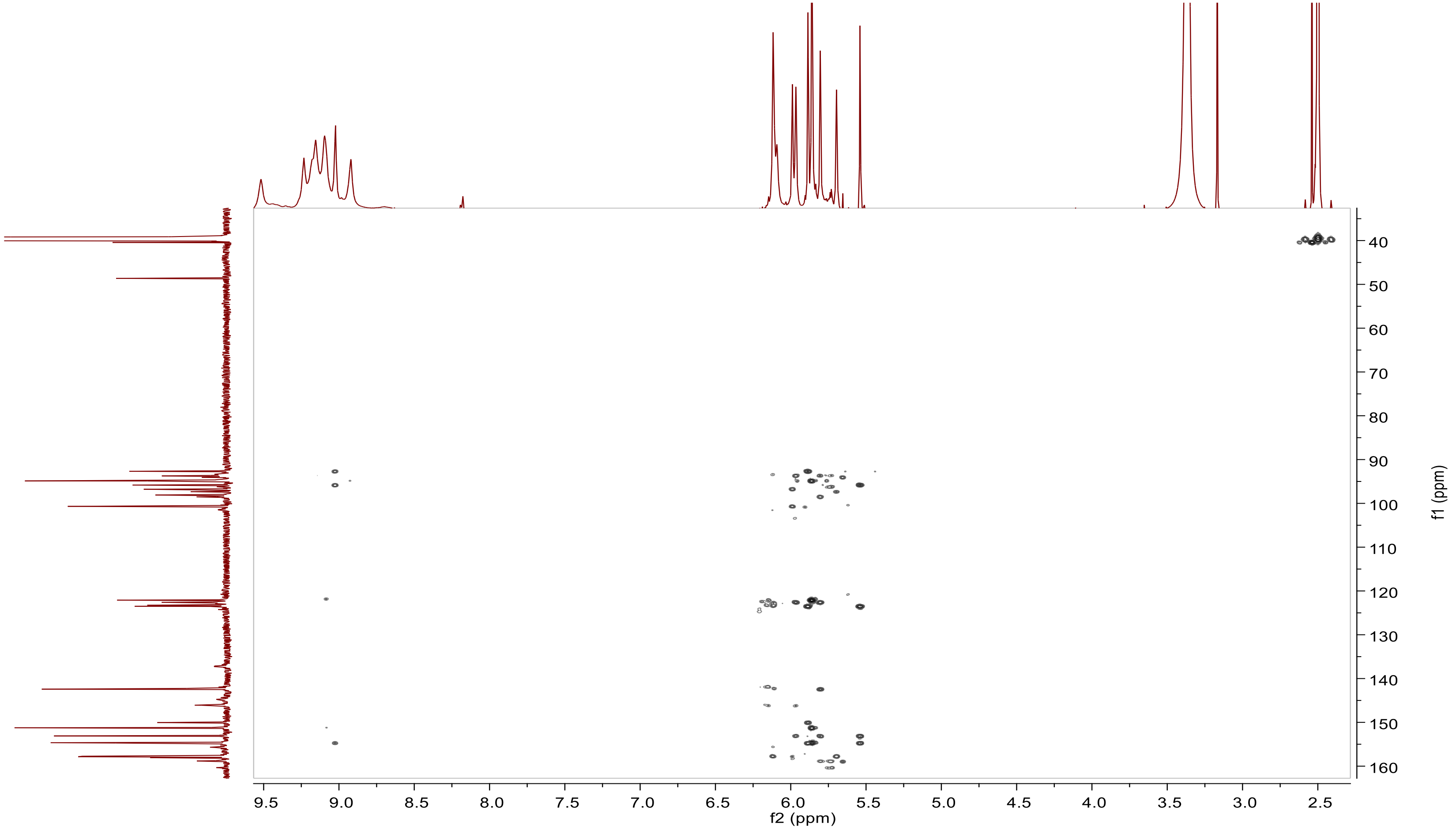

Figure S7. ROESY spectrum of compound **1** (800 MHz, DMSO-d_6).

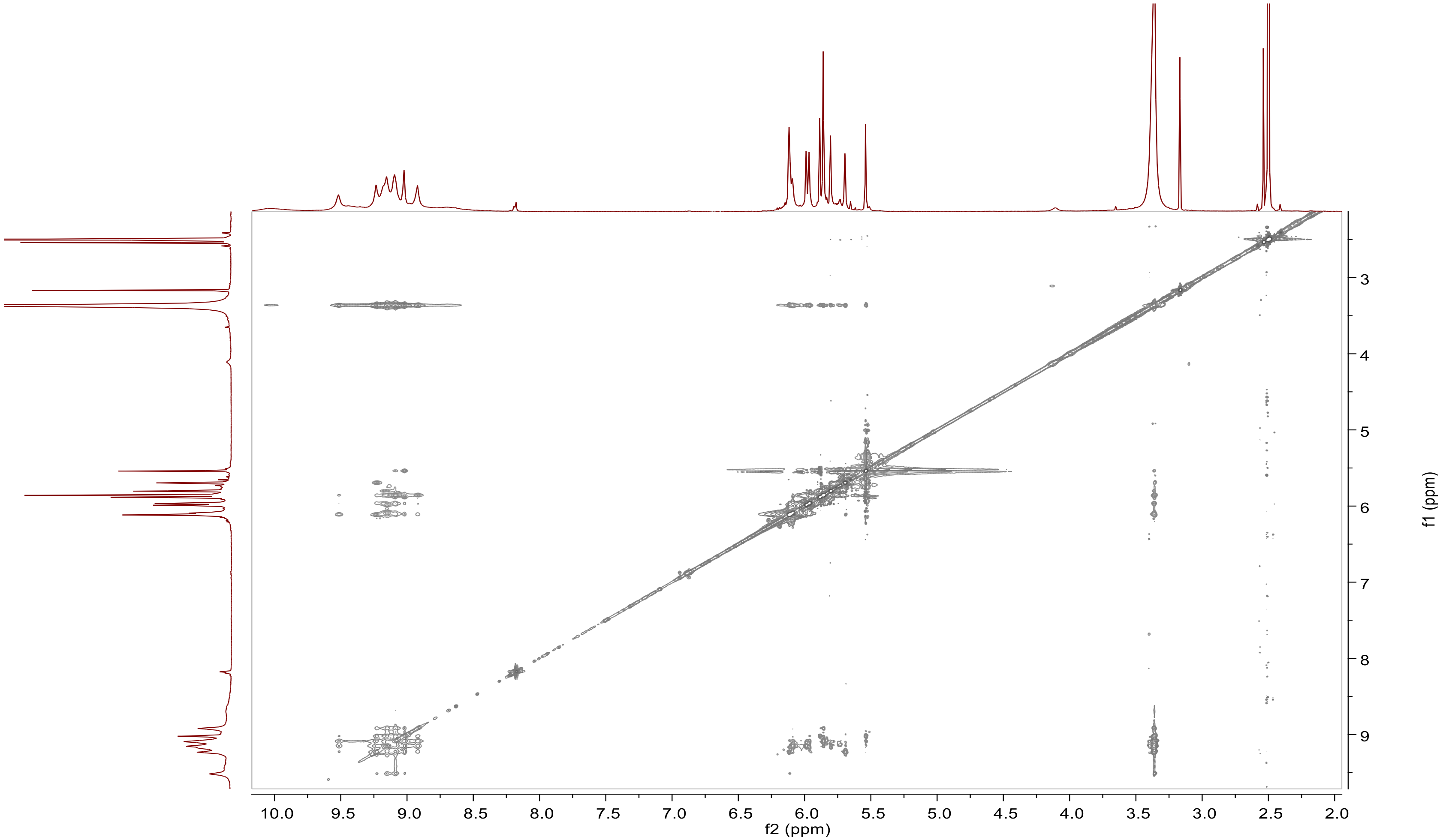

Figure S8. HRESIMS spectrum of compound **2.**

Qualitative Compound Report

**Data File**	EC2-3-2-6_5.d	**Sample Name**	EC2-3-2-6
**Sample Type**	Sample	**Position**	p1f1
**Instrument Name**	Instrument 1	**User Name**	6530-PC\admin
**Acq Method**	DIP method-0p.m	**Acquired Time**	11/8/2018 9:50:42 AM
**IRM Calibration Status**	Success	**DA Method**	Default.m
**Comment**			

Sample Group **Info.**

Stream Name LC 1

Compound Table

Compound Label	RT	Mass	Abund	Formula	Tgt Mass	Diff (ppm)
Cpd 1: C54H34O27	0.166	1114.1285	41944	C54H34O27	1114.1287	-0.18

Compound Label	RT	Algorithm	Mass
Cpd 1: C54H34O27	0.166	Find By Formula	1114.1285

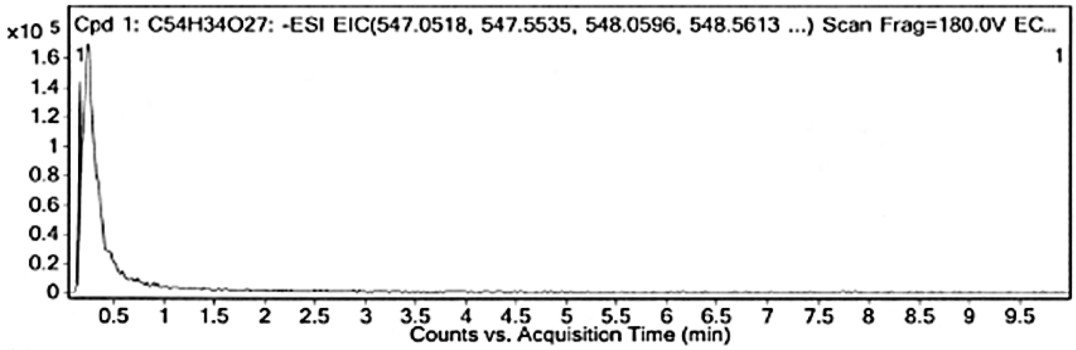

MS Spectrum

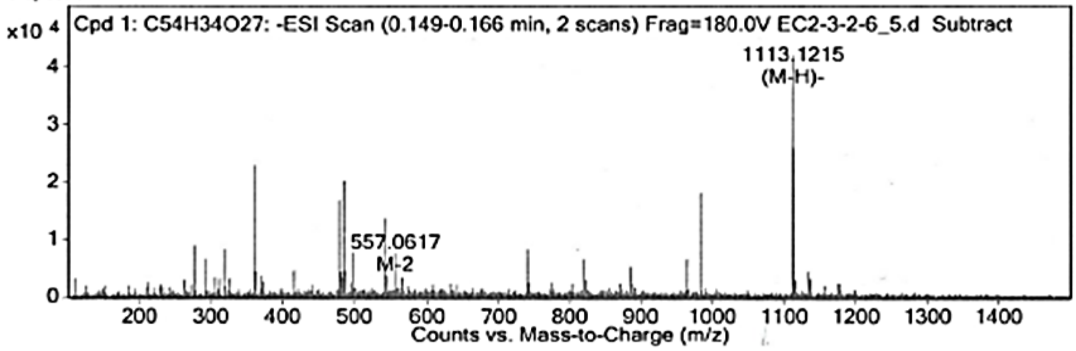

MS Spectrum Peak List

m/z	*Calc m/z*	Diff(ppm)	z	Abund	Formula	Ion
557.0617	557.0649	-5.71	-2	3347	C54 H34 O27	M-2
1095.0915	1095.1109	-17.76	-1	569	C54 H31 O26	(M-H)-[-H2O]
1096.1575	1096.1187	35.38	-1	248	C54 H32 O26	M*-[-H2O]
1113.1215	1113.1215	0.01		41944	C54 H33 O27	(M-H)-
1114.1237	1114.1249	-1.04		25939	C54 H33 O27	(M-H)-
1115.129	1115.1276	1.26		10241	C54 H33 O27	(M-H)-
1131.1	1131.0876	10.99	-1	60	C54 H32 Cl O26	(M+Cl)-[-H2O]
1141.1303	1141.1164	12.2	-1	302	C55 H33 O28	(M+HCOO)-[-H2O]
1149.1163	1149.0981	15.77	-1	624	C54 H34 Cl O27	(M+Cl)-
1159.1174	1159.1269	-8.28	-1	407	C55 H35 O29	(M+HCOO)-

--- End Of Report ---

Figure S9. IR spectrum of compound **2.**

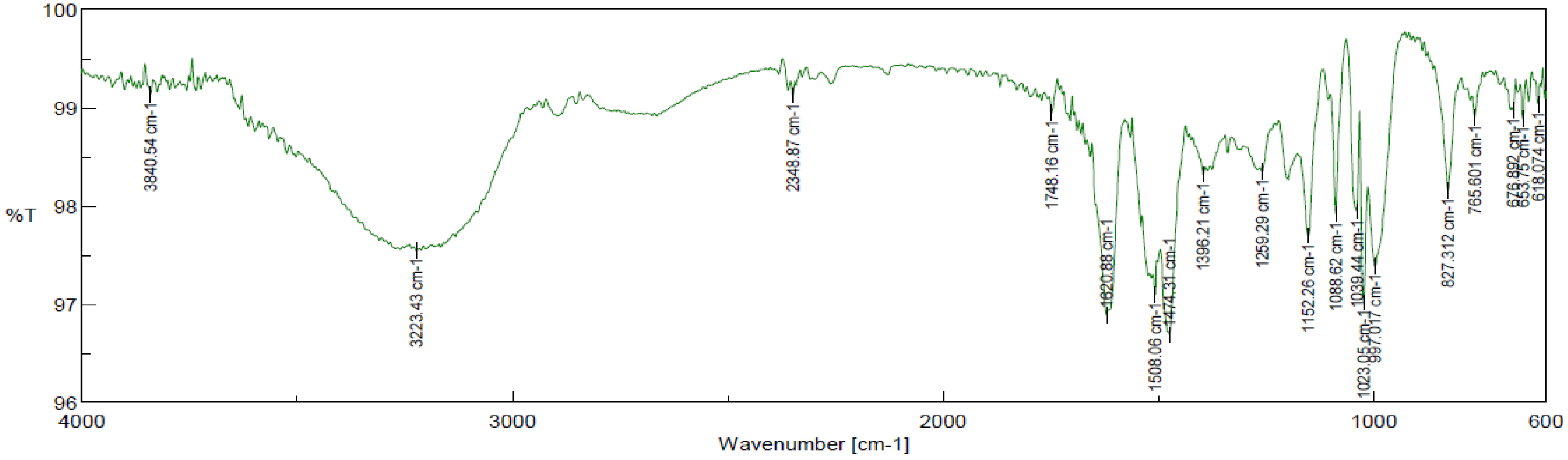

[Comment]	
Sample Name	
Comment	
User	
Division	
Company	공동기기실

[Data Information]	
Creation Date	2018-10-26 오전 11:10
Data array type	Linear data array
Horizontal	Wavenumber [cm-1]
Vertical	%T
Start	599.753 cm-1
End	4000.6 cm-1
Data pitch	0.964233 cm-1
Data points	3528

[Measurement Information]	
Model Name	FT/IR-4200typeA
Serial Number	B038361018
Light Source	Standard
Detector	TGS
Accumulation	16
Resolution	4 cm-1
Zero Filling	On
Apodization	Cosine
Gain	Auto (2)
Aperture	Auto (7.1 mm)
Scanning Speed	Auto (2 mm/sec)
Filter	Auto (30000 Hz)

Figure S10. ^{1}H NMR spectrum of compound **2** (850 MHz, DMSO-d_6).

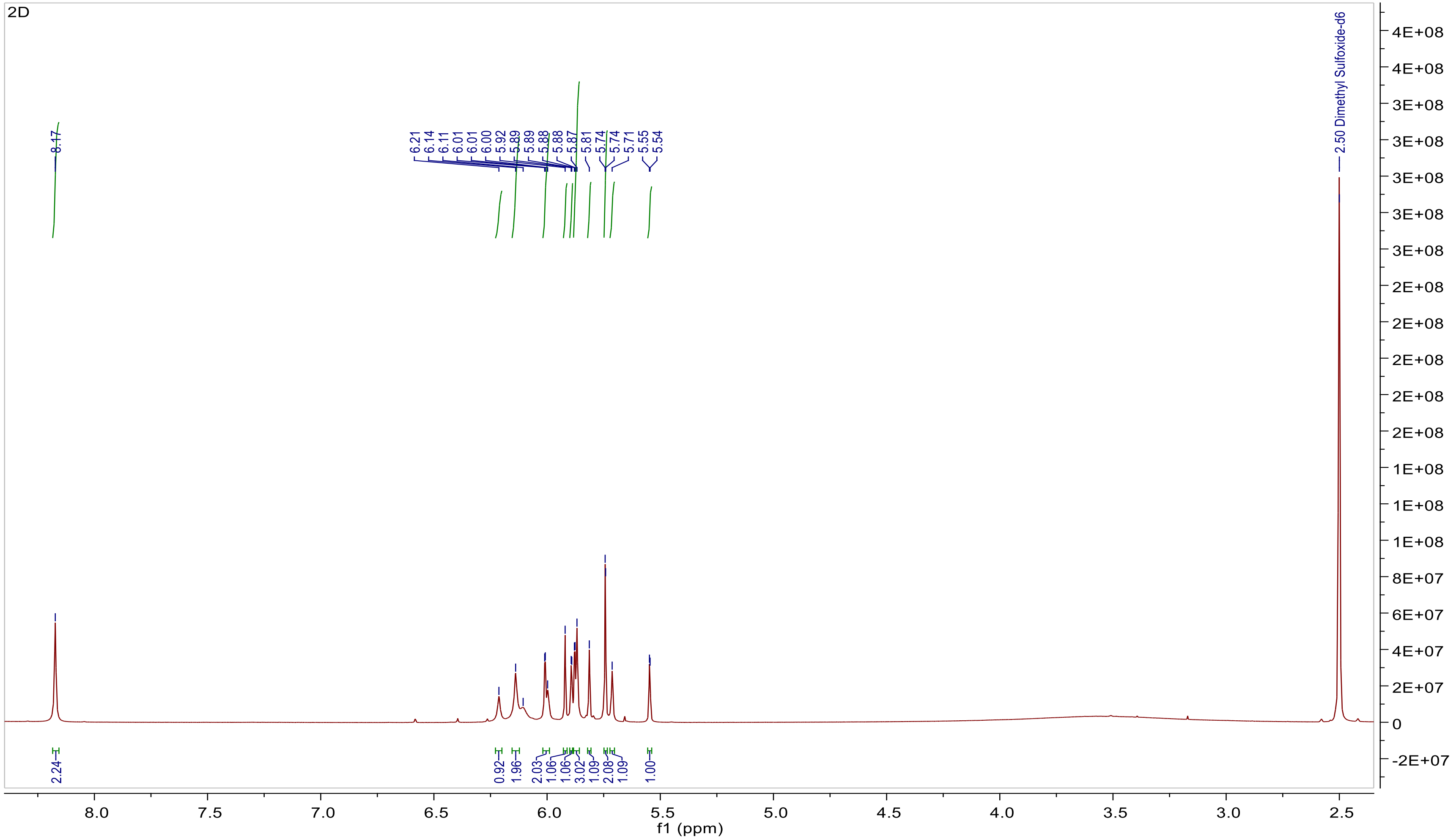

Figure S11. ^{13}C NMR spectrum of compound **2** (212.5 MHz, DMSO-d_6).

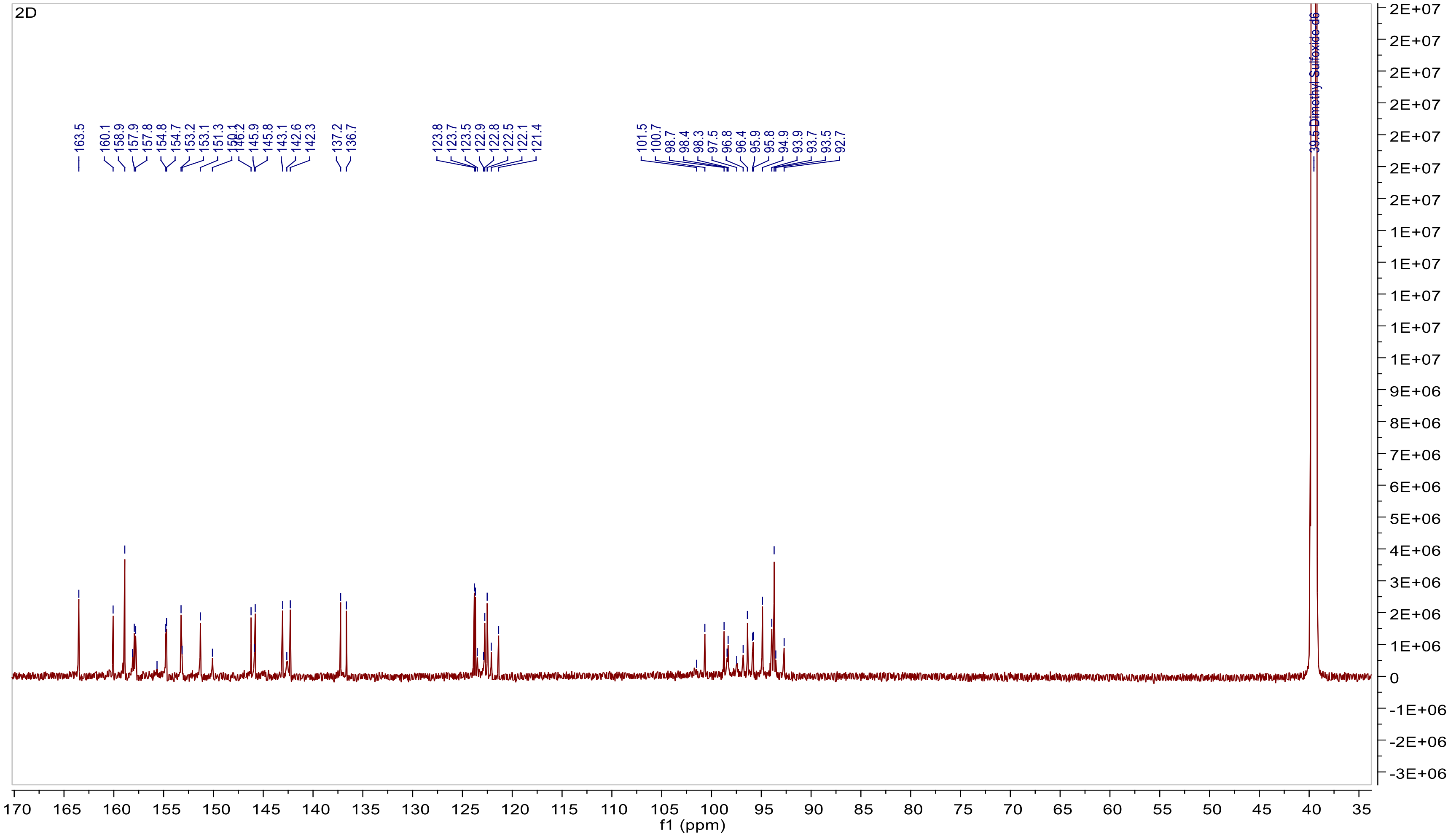

86 **Figure S12**. HSQC spectrum of compound **2** (850 MHz, DMSO-d_6).

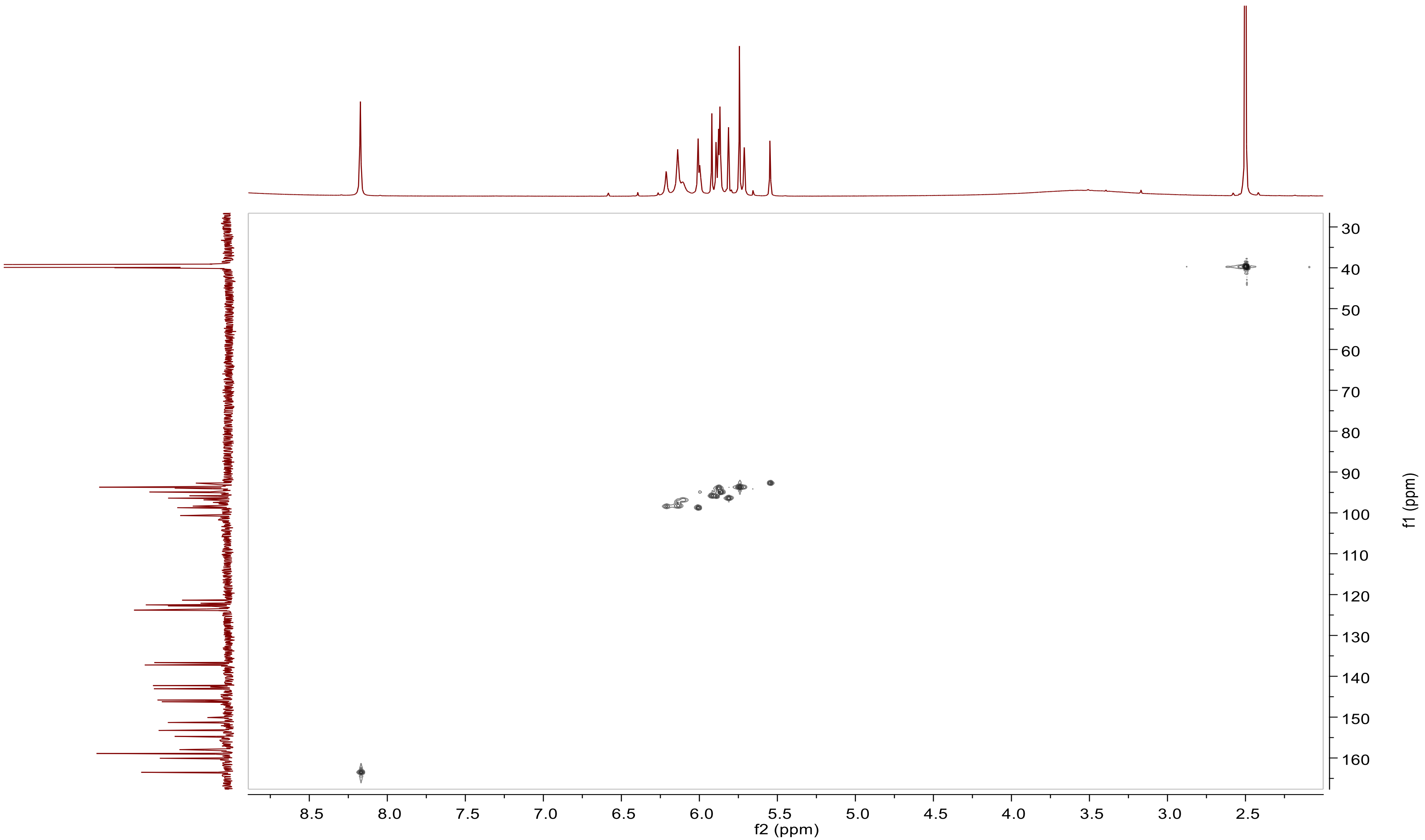

87

88 **Figure S13**. HMBC spectrum of compound **2** (850 MHz, DMSO-d_6).

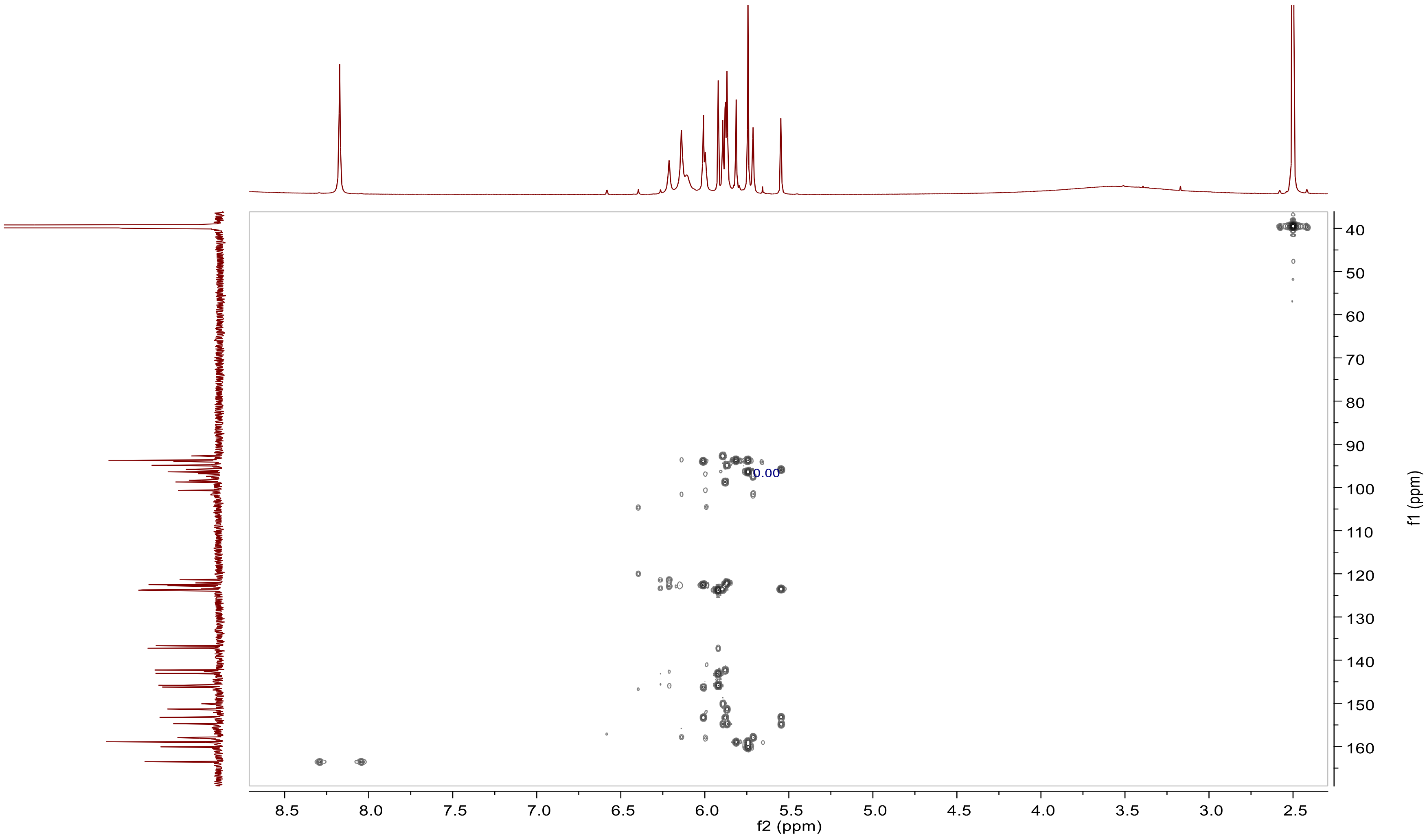

89

90 **Figure S14**. ROESY spectrum of compound **2** (850 MHz, DMSO-d_6).

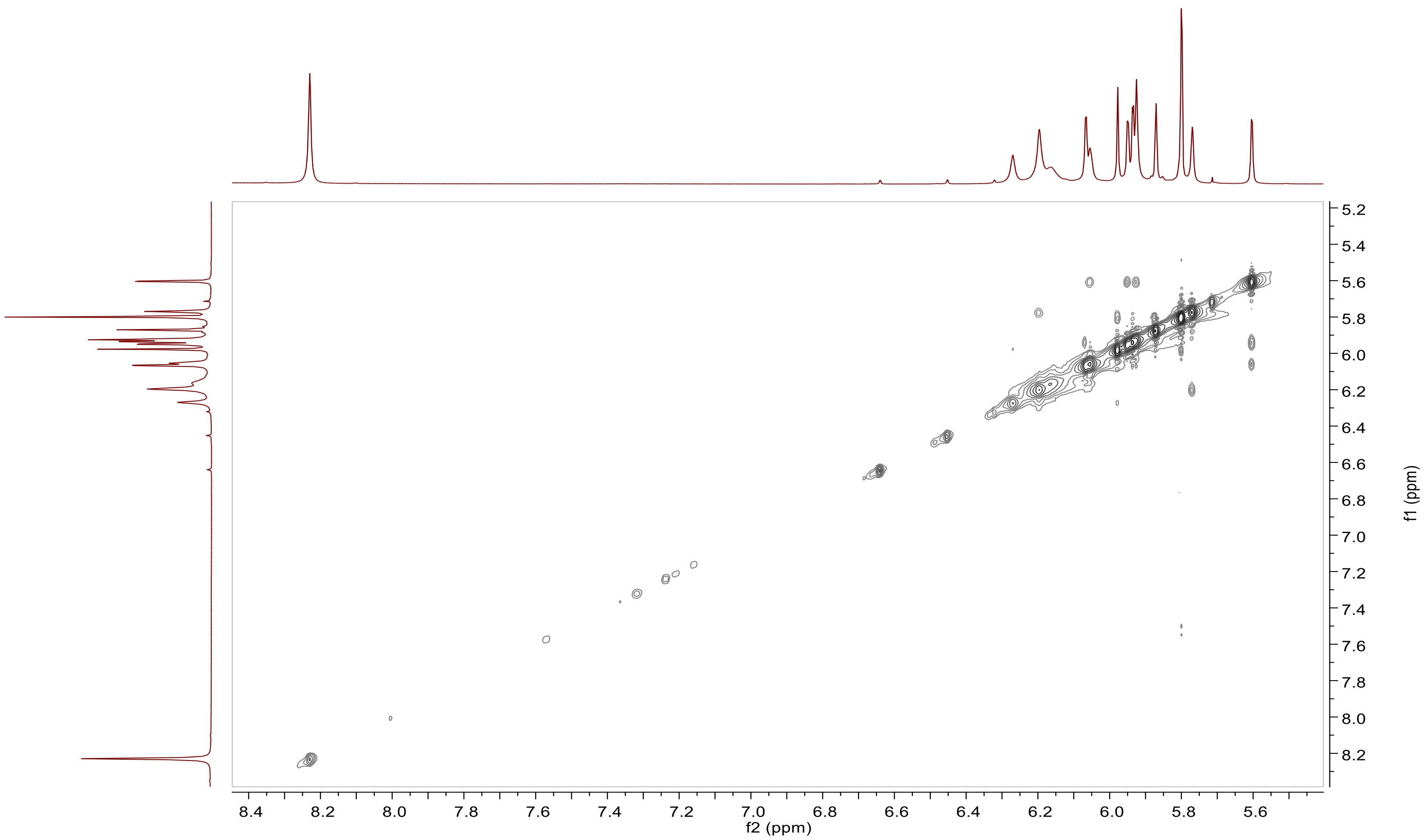

91

Figure S15. Relationships between ion masses (*m/z* value) in negative ion mode and RMD values for compounds detected by HPLC-qTOFMS in the EC70 fraction.

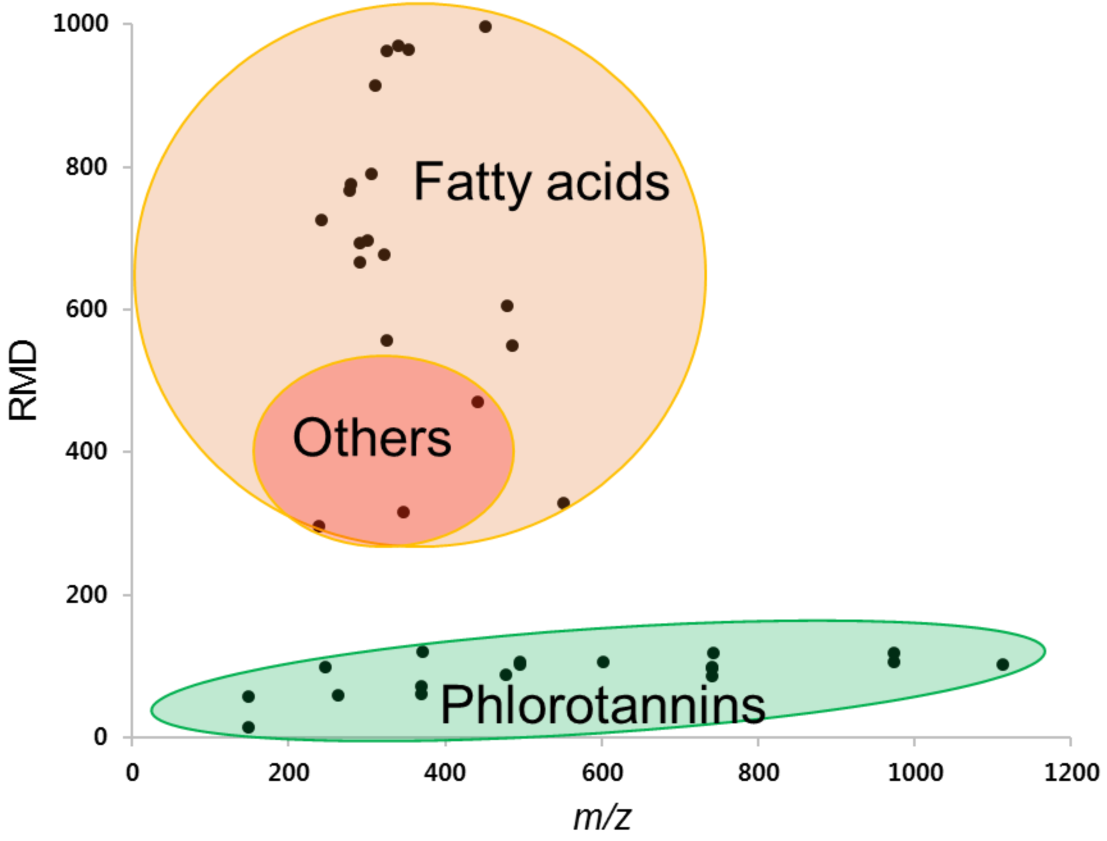

Figure S16. HPLC-qTOFMS measurement of twelve isolated compounds in negative ion mode at collision energy of 50 eV.

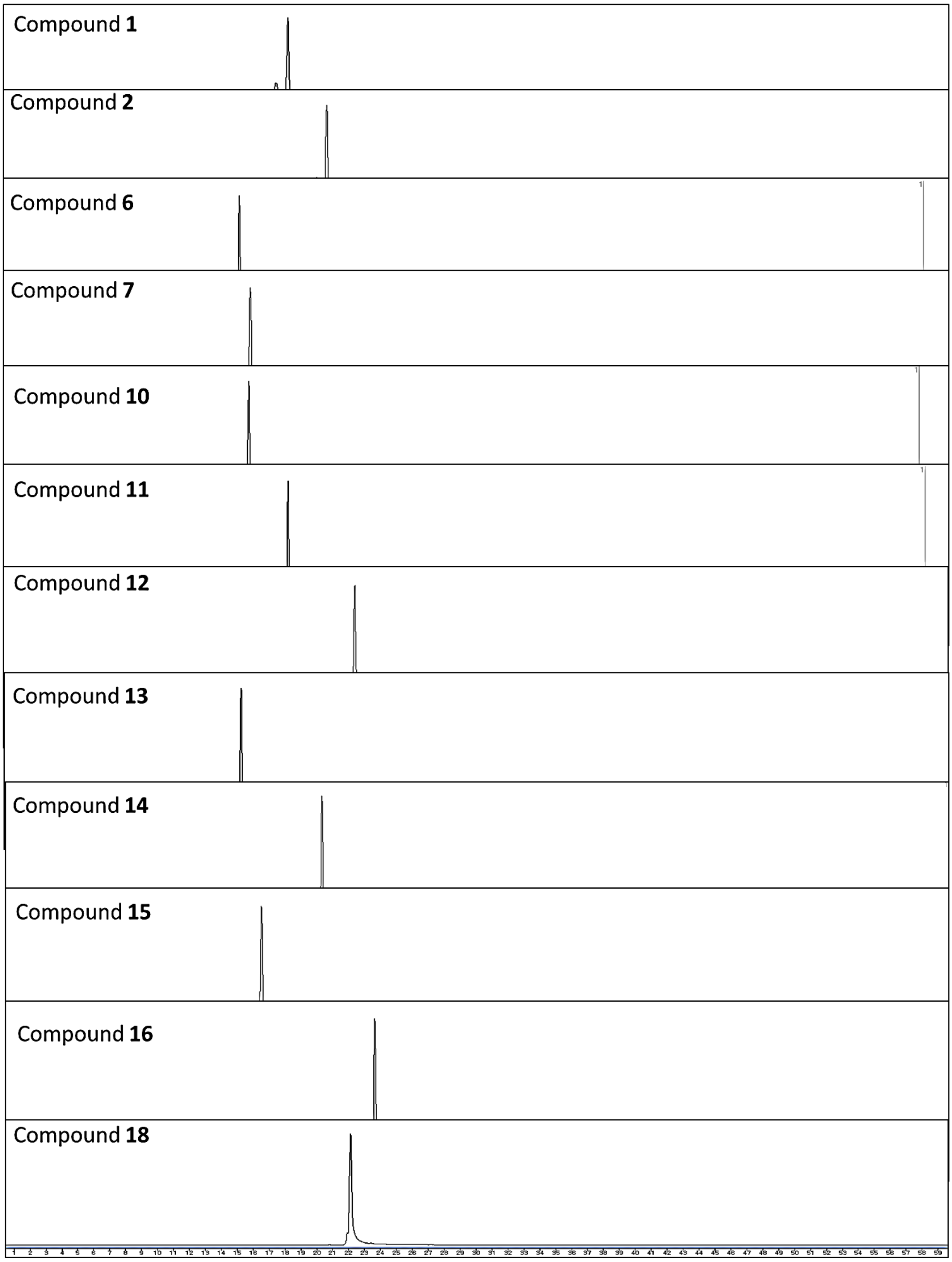

Figure S17. MS/MS spectra and fragment ions analysis of isolated compounds. HPLC-qTOFMS spectroscopic data of these single compounds were measured in negative mode at collision energy of 50 eV.

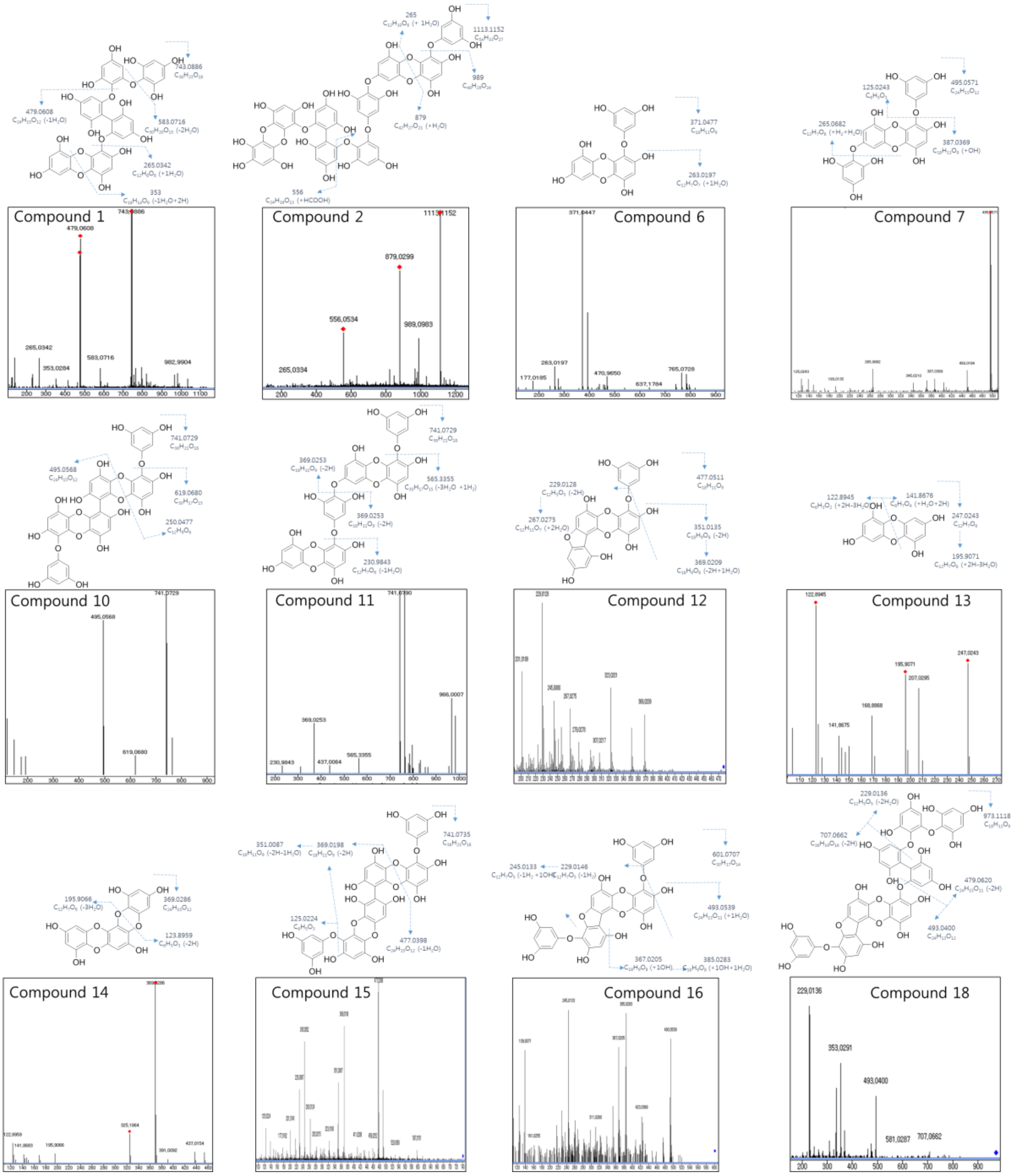

Figure S18. Effects of compound **11**, **12**, **13**, and **14** on the viral protein synthesis at a concentration of 20 µM; original uncropped blots.

The viral-infected cells were exposed to compounds **11**, **12**, **13**, and **14** (20 µM) and ribavirin (20 µM) as a positive control. After 1 day of incubation, the cell lysates were collected and target proteins were measured using Western blotting method. Equal amounts of proteins were loaded on SDS-polyacrylamide gels. After transferred to PVDF membranes, they were firstly incubated with neuraminidase antibody overnight. For removing bound primary and secondary antibodies, the membranes were incubated with a Restore™ Western blot stripping buffer (Thermo Sci.) and they were then detected the β-actin protein using a LAS4000 luminescent image analyzer. Sample names were from 1-7 as follows: uninfected ctrl, viral-infected (vi) ctrl, vi + ribavirin (20 µM), vi + **11**, **12**, **13**, and **14** (20 µM), respectively. Data were included in the final analysis in Figure 4A.

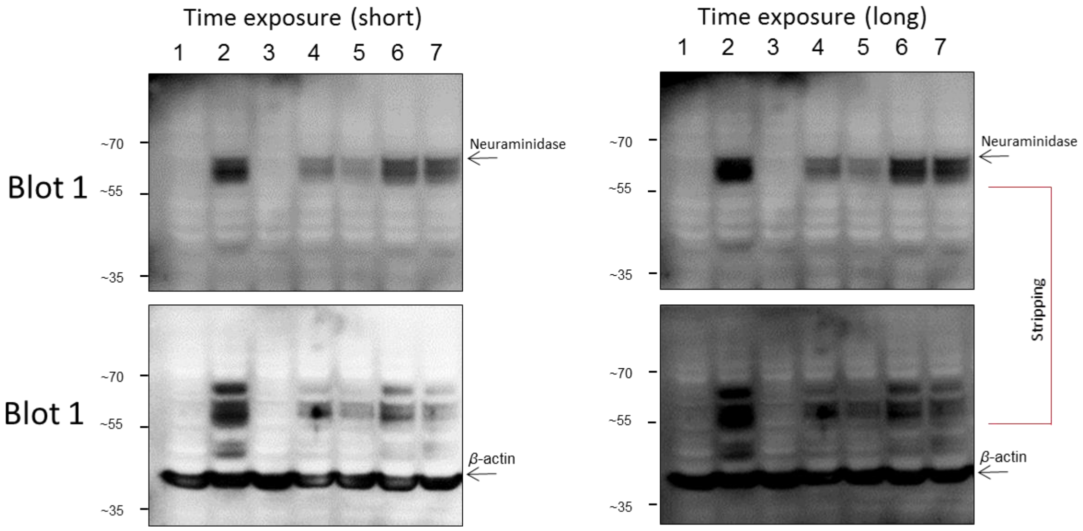

Figure S179. Inhibitory effects of compound **12** on the viral proteins synthesis in a concentration-dependent manner; original uncropped blots.

The procedure of Western blot experiment was successfully carried as above described in Figure S18. The membranes were firstly detected neuraminidase or hemagglutinin proteins. Using a stripping buffer (Thermo Sci.) for removing bound primary and secondary antibodies, the membranes were then incubated with β-actin antibody. (A) Sample names were from 1-7 as follows: uninfected ctrl, viral-infected (vi) ctrl, vi + ribavirin (20 μM), vi + **12** (5, 10, 20, and 40 μM), respectively. (B) Sample names were from 1-6 as follows: uninfected ctrl, vi ctrl, vi + ribavirin (20 μM), vi + **12** (5, 10, and 20 μM), respectively. (C) Sample names were from 1-12 as follows: uninfected ctrl-1; vi ctrl-1; vi + ribavirin (20 μM)-1, -2; vi + **12** (10 μM)-1, -2; vi + **12** (20 μM)-1, -2; vi + **12** (40 μM)-1, -2; vi ctrl-2; uninfected ctrl-2; respectively. Figure 4B was included in the final analysis.

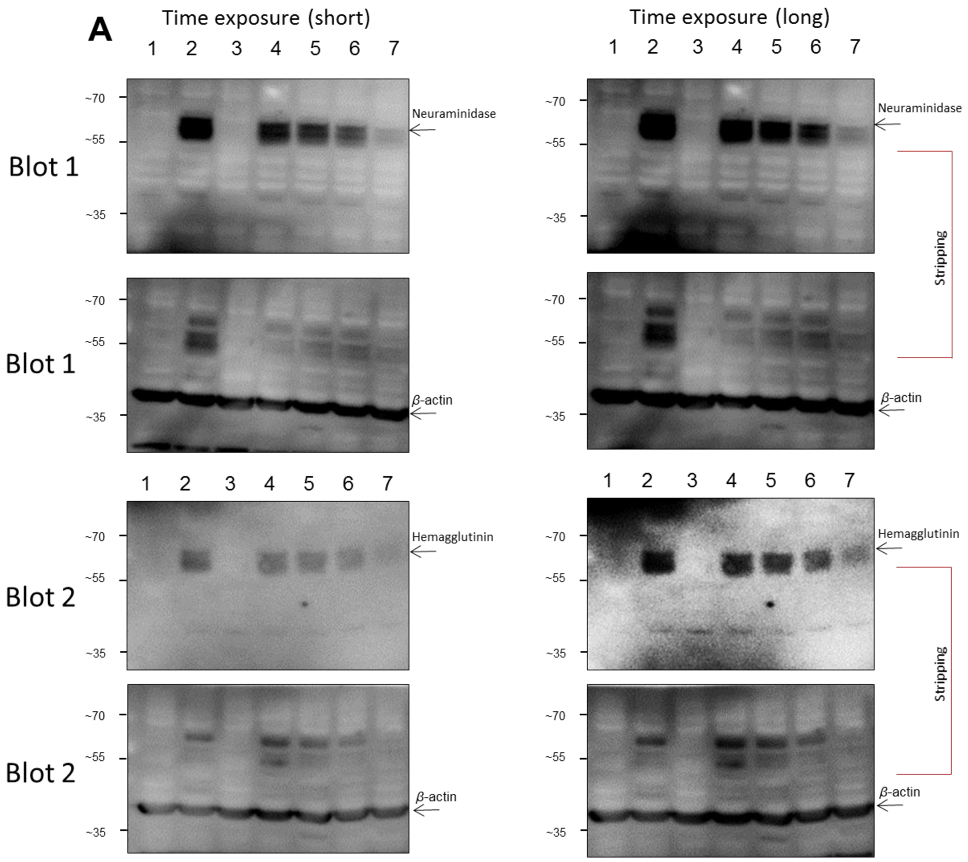

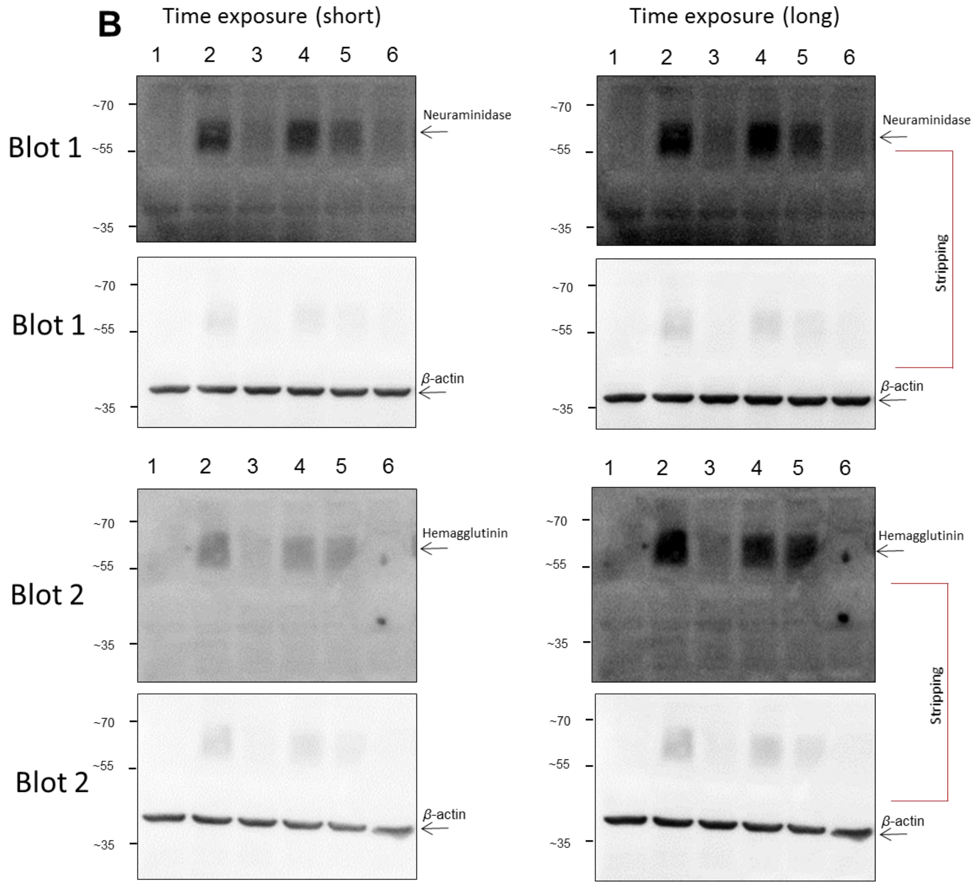
B
Time exposure (short)
Time exposure (long)
1 2 3 4 5 6
Blot 1
Blot 2
~70
~55
~35
Neuraminidase
Hemagglutinin
β-actin
Stripping

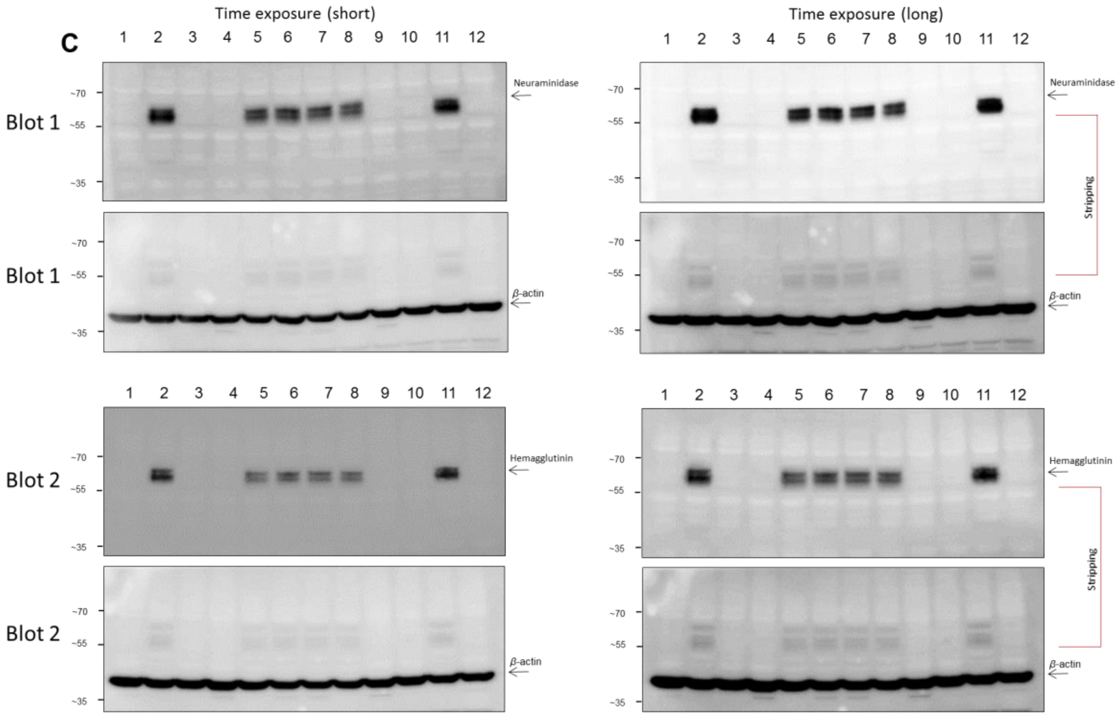
C
Time exposure (short)
Time exposure (long)
1 2 3 4 5 6 7 8 9 10 11 12
Blot 1
Blot 2
~70
~55
~35
Neuraminidase
Hemagglutinin
β-actin
Stripping

.

Figure S20. (A) Key HMBC correlations of compounds **1** and **2**. (B) Key ROESY correlations on 3D structure of compounds **1** and **2.**

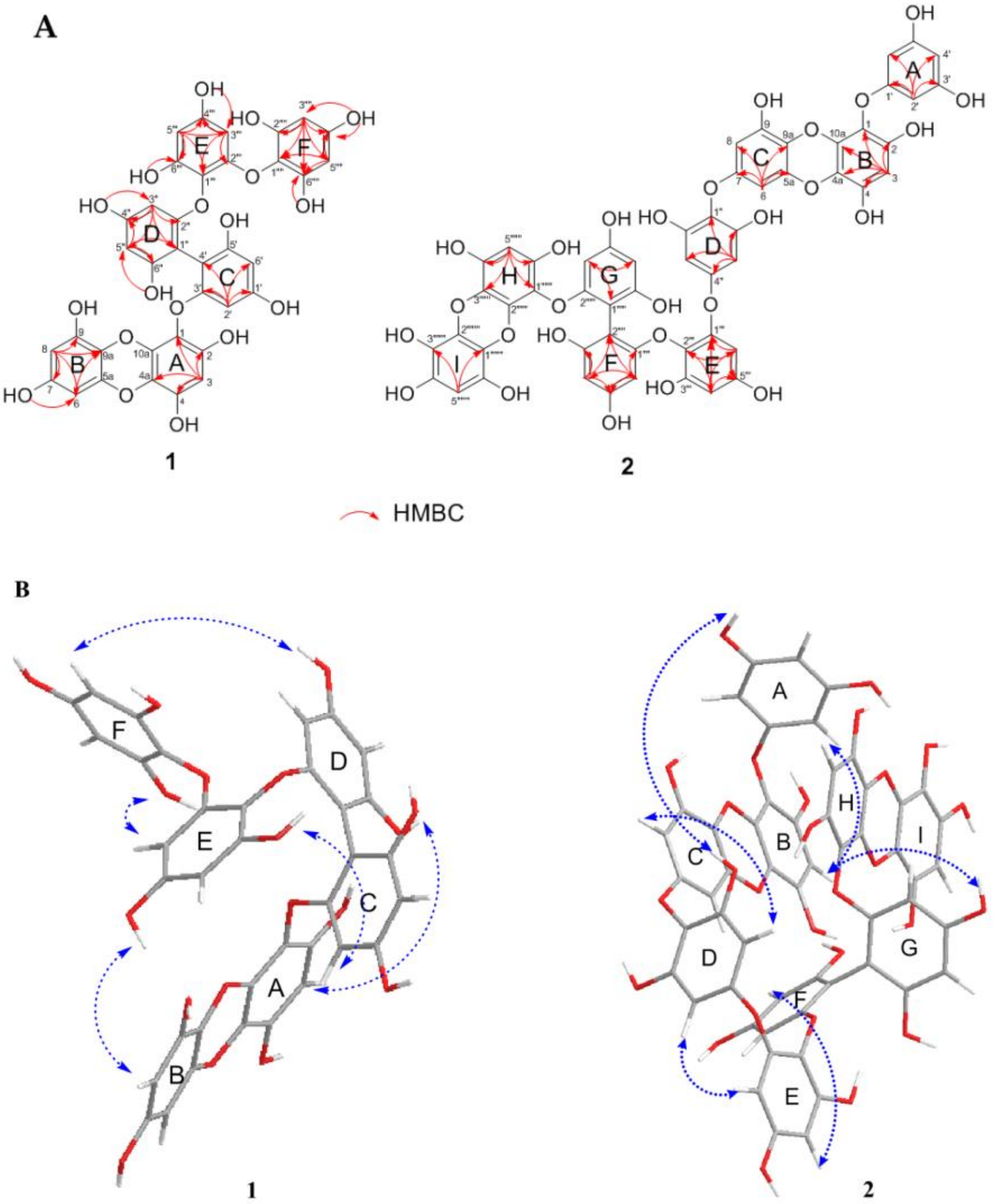

Figure S21. Physicochemical properties of isolated known compounds from *E. cava*.

Eckol (**6**)

Brown powder; UV (MeOH) λ_{max} nm (log ε) 210 (2.70), 235 (2.75); ESI-MS *m/z* 371 $[M - H]^-$; ^1H NMR (DMSO-d_6, 300 MHz): δ 6.16 (1H, s, H-3), 5.97 (1H, d, *J* = 2.4 Hz, H-8), 5.80 (1H, d, *J* = 2.4 Hz, H-6), 5.79 (1H, t, *J* = 2.4 Hz, H-4'), 5.71 (2H, d, *J* = 2.1 Hz, H-2', H-6'); ^{13}C NMR (DMSO-d_6, 75 MHz): δ 160.3 (C-1'), 158.8 (C-3', C-5'), 153.0 (C-7), 146.1 (C-9), 146.0 (C-2), 142.5 (C-5a), 141.9 (C-4), 137.1 (C-10a), 123.1 (C-1), 122.5 (C-9a), 122.0 (C-4a), 98.4 (C-8), 98.1 (C-3), 96.2 (C-4'), 93.7 (C-6), 93.6 (C-2', C-6').

7-Phloroeckol (**7**)

Brown powder; UV (MeOH) λ_{max} nm (log ε) 210 (2.34), 230 (2.27); ESI-MS *m/z* 495 $[M - H]^-$; ^1H NMR (DMSO-d_6, 300 MHz): δ 6.14 (s, H-3), 6.00 (1H, d, *J* = 2.9, H-8), 5.77 (1H, d, *J* = 2.9, H-6), 5.80 (1H, d, *J* = 1.9 Hz, H-4'), 5.86 (1H, d, *J* = 2.4 Hz, H-2', H-6'); ^{13}C NMR (DMSO-d_6, 75 MHz): δ 122.2 (C-1), 146.0 (C-2), 98.4 (C-3), 141.9 (C-4), 123.2 (C-4a), 142.9 (C-5a), 93.4 (C-6), 154.6 (C-7), 98.1 (C-8), 146.1 (C-9), 124.0 (C-9a), 137.1 (C-10a), 160.3 (C-1'), 93.6 (C-2'), 159.0 (C-3'), 96.3 (C-4'), 159.0 (C-5'), 93.7 (C-6'), 122.9 (C-1''), 151.3 (C-2''), 94.9 (C-3''), 154.9 (C-4''), 94.9 (C-5''), 151.3 (C-6'')

6,6'-Bieckol (**10**)

Brown powder; UV (MeOH) λ_{max} nm (log ε) 210 (2.34), 230 (2.27); ESI-MS *m/z* 743 $[M + H]^+$, 741 $[M - H]^-$; ^1H NMR (DMSO-d_6, 300 MHz): δ 6.09 (s, H-3), 6.04 (1H, s, H-8), 5.80 (1H, d, *J* = 1.8 Hz, H-4'), 5.74 (1H, d, *J* = 2.1 Hz, H-2', H-6'); ^{13}C NMR (DMSO-d_6, 75 MHz): δ 160.5 (C-1'), 158.9 (C-3', C-5), 151.3 (C-7), 145.4 (C-2), 144.5 (C-9), 141.9 (C-

4), 141.4 (C-5a), 137.2. (C-10), 123.6 (C-1), 122.7 (C-9a), 122.0 (C-4a), 99.7 (C-6), 97.8 (C-8), 97.8 (C-3), 96.2 (C-4'), 93.7 (C-2', C-6').

Dieckol (**11**)

Brown powder; UV (MeOH) λ_{max} nm (log *ε*) 210 (2.70), 235 (2.75); ESI-MS *m/z* 743 [M + H]$^+$, 741 [M − H]$^-$; ^{1}H NMR (DMSO-d_6, 300 MHz): *δ* 6.16 (1H, s, H-1"), 6.14 (1H, s, H-3), 6.01 (1H, d, *J* = 2.2 Hz, H-8), 6.99 (1H, d, *J* = 2.2 Hz, H-8"), 5.94 (1H, s, H-2"', H-6"'), 5.82 (1H, br d, *J* = 2.7 Hz, H-6), 5.81 (1H, br d, *J* = 2.8 Hz, H-6"), 5.79 (1H, t-like, H-4'), 5.71 (1H, br d, H-2', H-6'); ^{13}C NMR (DMSO-d_6, 75 MHz): *δ* 160.3 (C-1'), 158.8 (C-2', C-5'), 155.9 (C-1"'), 154.2 (C-7), 153.1 (C-7"), 151.2 (C-3"', C-5"'), 146.1 (C-2), 146.1 (C-9"), 146.0 (C-9), 145.9 (C-2"), 142.6 (C-5a"), 142.4 (C-5a), 142.0 (C-4"), 141.9 (C-4), 137.2 (C-10a), 137.0 (C-10a"), 124.2 (C-1"), 124.0 (C-9a), 123.2 (C-4a") 123.1 (C-4), 122.5 (C-9a"), 122.2 (C-1"), 122.1 (C-1).

Phlorofucofuroeckol A (**12**)

Brown powder; UV (MeOH) λ_{max} nm (log *ε*) 210 (3.17), 225 (3.16); ESI-MS *m/z* 601 [M − H]$^-$; ^{1}H NMR (DMSO-d_6, 300 MHz): *δ* 6.72 (1H, s, H-13), 6.43 (1H, s, H-9), 6.30 (1H, s, H-3), 5.83 (2H, br t, *J* = 1.6 Hz, H-4', H-4"), 5.76 (2H, d, *J* = 2.0 Hz, H-2', H-6'), 5.72 (2H, d, *J* = 2.0 Hz, H-2', H-6'); ^{13}C NMR (DMSO-d_6, 75 MHz): *δ* 160.2 (C-1'), 160.0 (C-1"), 159.0 (C-3", C-5"), 158.9 (C-3', C-5'), 150.8 (C-12a), 150.4 (C-10), 149.5 (C-11a), 147.0 (C-2), 146.5 (C-8), 144.8 (C-14), 142.1 (C-4), 136.8 (C-15a), 134.0 (C-5a), 126.3 (C-14a), 122.5 (C-1), 122.4 (C-4a), 120.1 (C-11), 103.4 (C-7), 103.2 (C-6), 99.1 (C-9), 98.3 (C-3), 96.3 (C-4, C-4"), 94.8 (C-13), 93.7 (C-2', C-6'), 93.5 (C-2", C-6").

Dibenzo [1,4]dioxine-2,4,7,9-tetraol (**13**)

Brown powder; UV (MeOH) λ_{max} nm (log ε) 228 (1.59), 278 (0.79); ESI-MS *m/z* 247 [M − H]$^{-}$; ^{1}H NMR (DMSO-d_6, 300 MHz): δ 5.96 (2H, d, J = 2.7 Hz, H-3, H-8), 5.78 (2H, d, J = 2.7 Hz, H-1, H-6); ^{13}C-NMR (DMSO-d_6, 75 MHz): δ 152.8 (C-2, C-7), 145.8 (C-4, C-9), 142.8 (C-5a, C-10), 122.9 (C-4a C-9a), 98.3 (C-3, C-8), 93.9 (C-1, C-6).

Dioxinodehydroeckol (**14**)

Brown powder; UV (MeOH) λ_{max} nm (log ε) 235 (2.85); ESI-MS *m/z* 369 [M − H]$^{-}$; ^{1}H NMR (DMSO-d_6, 300 MHz): δ 6.10 (1H, s, H-7), 6.05 (1H, d, J = 2.7 Hz, H-2), 6.02 (1H, d, J = 2.7 Hz, H-10), 5.84 (1H, d, J = 2.7 Hz, H-4), 5.82 (1H, d, J = 2.7 Hz, H-12); ^{13}C NMR (DMSO-d_6, 75 MHz): δ 153.3 (C-3), 153.0 (C-11), 146.1 (C-1), 145.9 (C-9), 142.0 (C-4a), 141.7 (C-12a), 140.1 (C-6), 137.1 (C-7a), 131.5 (C-13b), 122.6 (C-8), 122.5 (C-13a), 122.2 (C-14a), 98.8 (C-2, C-10), 97.9 (C-7), 93.9 (C-4, C-12).

6,8’-Bieckol (**15**)

Brown powder; UV (MeOH) λ_{max} nm (log ε) 210 (2.34), 230 (2.27); ESI-MS *m/z* 743 [M + H]$^{+}$, 741 [M − H]$^{-}$; ^{1}H NMR (DMSO-d_6, 300 MHz): δ 6.13 (1H, s, H-8), 6.03 (1H, s, H-3'), 6.00 (1H, s, H-3), 5.92 (1H, s, H-6'), 5.75 (2H, t, J = 1.6 Hz, H-4'', H-4'''), 5.71 (2H, d, J = 2.4 Hz, H-2''', H-6'''), 5.68 (2H, J = 1.6 Hz, H-2'', H-6''); ^{13}C NMR (DMSO-d_6, 75 MHz): δ 160.0(C-1'', C-1'''), 158.0 (C-3''', C-5'''), 158.0 (C-3'', C-5''), 151.5 (C-7, C-7'), 145.8 (C-2), 145.5 (C-2'), 144.9 (C-9), 144.4 (C-9'), 141.9 (C-4), 141.4 (C-4'), 141.7 (C-5a), 140.0 (C-5a'), 137.3 (C-10a), 137.2 (C-10a'), 123.6 (C-1), 123.4 (C-1'), 123.0 (C-9a), 122.9 (C-9a'), 122.4 (C-4a), 122.0 (C-4a'), 104.5 (C-8'), 99.5 (C-6), 98.2 (C-8), 97.0 (C-3), 97.0 (C-3'), 96.3 (C-4''), 93.9 (C-2''', C-6'''), 93.8 (C-2'', C-6''), 93.7 (C-6').

Fucofuroeckol A (**16**)

Brown powder; UV (MeOH) λ_{max} nm (log ε) 208 (2.10), 224 (2.33), 300 (1.63); ESI-MS *m/z* 477 $[M - H]^-$; ^{1}H NMR (DMSO-d_6, 300 MHz) δ 6.72 (1H, s), 6.47 (1H, s), 6.29 (1H, s), 6.25 (1H, d, J = 1.8 Hz, H-9), 5.83 (1H, t, J = 2.1 Hz, H-4'), 5.76 (2H, d, J = 2.1 Hz, H-2', H-6')

974-A (**18**)

Brown powder; UV (MeOH) λ_{max} nm (log ε) 208 (2.10), 224 (2.33), 300 (1.63); ESI-MS *m/z* 973 $[M - H]^-$; ^{1}H NMR (DMSO-d_6, 300 MHz) δ 6.15 (1H, d, J = 2.1 Hz, H-4'), 6.01 (1H, d, J = 2.1 Hz, H-6'), 6.26 (1H, s, H-3), 6.72 (1H, s, H-6), 6.44 (1H, s, H-10), 6.73 (2H, d, J = 1.8 Hz, H-2'', H-6''), 5.83 (1H, dt, J = 1.8, 2.1 Hz, H-4''), 6.11 (1H, d, J = 1.2 Hz, H4'''), 5.75 (1H, d, J = 1.5 Hz, H-6'''), 5.86 (2H, s, H-3''''', H-5''''') ; ^{13}C-NMR (DMSO-d_6, 75 MHz): δ 157.9 (C-1'), 120.1 (C-2'), 157.7 (C-3'), 99.2 (C-4'), 158.8 (C-5'), 95.9 (C-6'), 136.9 (C-1), 146.6 (C-2), 103.4 (C-3), 146.1 (C-4), 141.9 (C-4a), 142.4 (C-15a), 145.9 (C-5a), 92.7 (C-6), 153.1 (C-6), 153.1 (C-6a), 123.5 (C-13), 123.5 (C-13), 144.7 (C-14), 142.0 (C-14a), 150.1 (C-7a), 126.4 (C-8), 150.4 (C-9), 103.3 (C-10), 149.6 (C-11), 122.9 (C-12), 160.3 (C-1''), 96.5 (C-2'', C-6''), 160.0 (C-3'', C-5''), 98.4 (C-4''), 158.1 (C-1'''), 122.1 (C-2'''), 157.8 (C-3'''), 100.7 (C-4'''), 159.0 (C-5'''), 94.9 (C-6'''), 137.1 (C-1''''), 151.3 (C-2''''), 97.5 (C-3''''), 155.8 (C-4''''), 93.6 (C-5''''), 154.7 (C-6''''), 133.9 (C-1'''''), 150.9 (C-2''''', C-6'''''), 96.9 (C-3''''', C-5'''''), 154.8 (C-4''''').

Table S1. List of spicies in *Ecklonia* genus from website World Register of Marine Species (http://www.marinespecies.org).

No	Species	Other name	Distribution
1	*Ecklonia bicyclis*	*Eisenia bicyclis*<br>*Ecklonia wrightii*	Pacific Ocean waters (Japan, Korea)
2	*Ecklonia biruncinata*	*Ecklonia exasperata,*<br>*Ecklonia radiata*	Indian Ocean (Australia, Madagascar, Oman, South Africa)
3	*Ecklonia brevipes*		Australia, New Zealand
4	*Ecklonia buccinalis*	*Ecklonia maxima*	South Africa
5	*Ecklonia caepaestipes*	*Durvillaea antarctica*	New Zealand
6	*Ecklonia cava*	*Ecklonia latifolia*	Japan, Korea
7	*Ecklonia fastigiata*		South Africa
8	*Ecklonia kurome*		Japan
9	*Ecklonia muratii*		Northern hemisphere (Mauritania, Senegal)
10	*Ecklonia radicosa*	*Eckloniopsis radicosa*	Japan
11	*Ecklonia richardiana*		New Zealand
12	*Ecklonia stolonifera*		Japan, Korea

Table S2. The inhibitory effects of compounds **11**, **12**, **13** and **14** against the H1N1 A/PR/8/34 virus in a cytopathic effect and cytotoxicity assays.

**Comp. No**	**EC_{50} (μM)**	**CC_{50} (μM)**
**11**	17.34 ± 3.97	> 100
**12**	13.48 ± 1.93	> 100
**13**	23.95 ± 2.00	> 100
**14**	23.41 ± 4.72	> 100
Ribavirin	4.29 ± 1.30	-

EC_{50}: Effective concentration; CC_{50}: Cytotoxic concentration

Table S3. ^{1}H and ^{13}C NMR spectroscopic data of compounds **1** and **2** in DMSO-d_6 (δ in ppm).

		1[a]			2[b]	
Ring	No.	δ_H, (Mult. *J* in Hz)	δ_C	No.	δ_H, (Mult. *J* in Hz)	δ_C
A	1		122.6	1'		160.1
	2		144.7	2', 6'	5.74 (2H, d, 1.8)	93.7
	3	6.12 (1H, s, overlap)	98.1	3', 5'		158.9
	4		142.2	4'	5.81 (1H, brt)	96.4
	4a		123.3			
	10a		137.2			
B	5a		142.4	1		123.7
	6	5.81 (1H, d, 2.7)	93.7	2		145.8
	7		153.1	3	5.92 (1H, s)	95.8
	8	5.97 (1H, d, 2.7)	98.5	4		143.1
	9		146.1	4a		123.8
	9a		122.6	10a		137.2
	OH-7	9.11 (1H, brs)				
C	1'		157.9	5a		142.3
	2'	5.99 (1H, d, 1.5)	94.7	6	5.88 (1H, d, 2.2)	93.9
	3'		157.7	7		153.2
	4'		100.7	8	6.01 (1H, d, 2.2)	98.7
	5'		158.1	9		146.2
	6'	6.09(1H, brs)	96.8	9a		122.5
D	1''		101.4	1''		122.1
	2''		155.7	2'', 6''		151.3
	3''	5.70 (1H, d, 1.5)	93.8	3'', 5''	5.87 (2H, s)	94.9
	4'', 6''		157.7	4''		154.7
	5''	6.12 (1H, s, overlap)	97.3			
	OH-4''	8.88 91H, brs)				
	OH-6''	9.19 (1H, brs)				
E	1'''		123.5	1'''		123.5
	2'''		150.0	2'''		150.1
	3'''	5.54 (1H, d, 2.7)	92.7	3'''	5.55 (1H, d, 2.4)	92.7
	4'''		153.1	4'''		153.1
	5'''	5.89 (1H, d, 2.7)	95.8	5'''	5.89 (1H, d, 2.4)	95.9
	6'''		154.7	6'''		154.8
	OH-4''', 6'''	8.98 (2H, brs)				
F	1''''		122.1	1''''		101.5
	2'''', 6''''		151.2	2''''		157.9
	3'''', 5''''	5.86 (2H, s)	94.9	3''''	5.71 (1H, brs)	93.5
	4''''		154.7	4''''		157.8
	OH-4''''	8.89 (1H, brs)		5''''	6.14 (1H, brs)	97.5
	OH-6''''	9.05 (1H, brs)		6''''		155.7
G				1'''''		100.7
				2'''''		
				3'''''	6.00 (1H, brs)	94.9
				4'''''		157.8
				5'''''	6.11 (1H, brs)	96.8
				6'''''		
H				1''''''		121.4
				2''''''		136.7
				3''''''		122.9
				4''''''		142.6
				5''''''	6.21 (1H, s)	98.4
				6''''''		145.9
I				1'''''''		
				2'''''''		
				3'''''''		
				4'''''''	6.14 (1H, s)	98.3
				5'''''''		
				6'''''''		

[a] measured in 800 MHz, [b] measured in 850 MHz

Scheme S1. Extraction and fractionation of *E. Cava*.

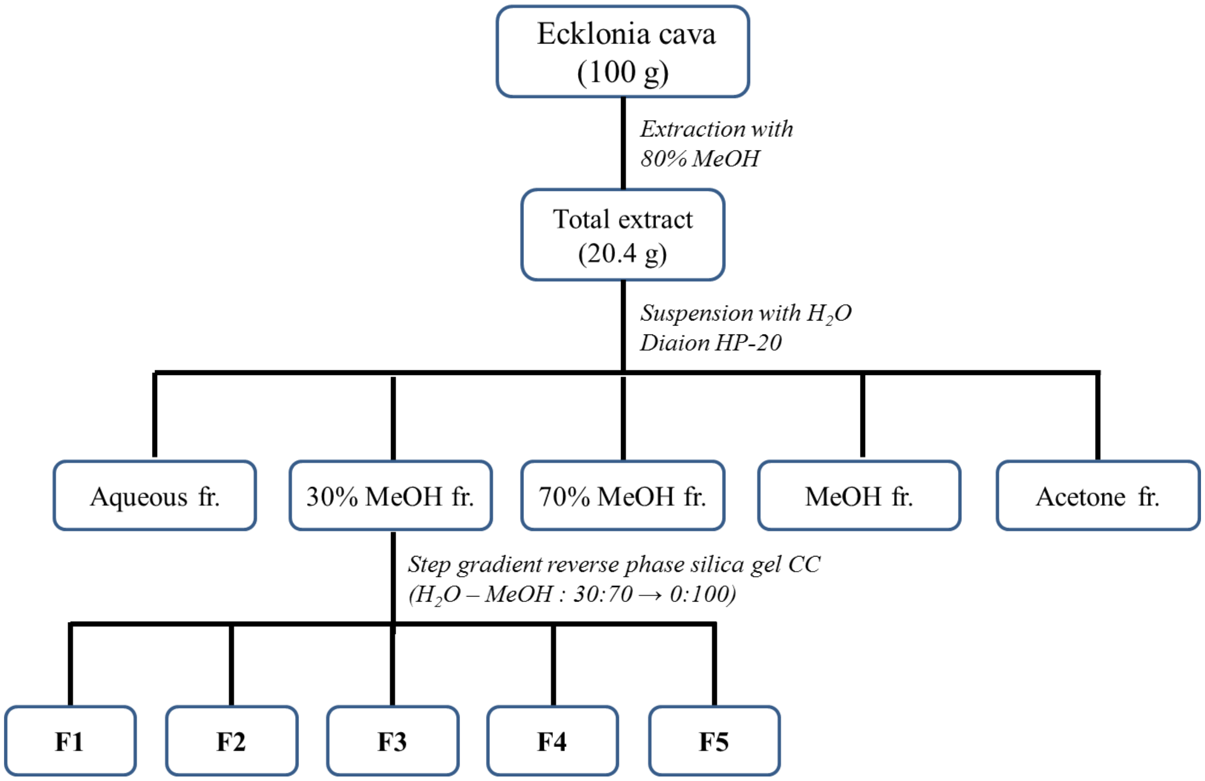
Ecklonia cava
(100 g)
Extraction with
80% MeOH
Total extract
(20.4 g)
Suspension with H_2O
Diaion HP-20
Aqueous fr.
30% MeOH fr.
70% MeOH fr.
MeOH fr.
Acetone fr.
Step gradient reverse phase silica gel CC
(H_2O – MeOH : 30:70 → 0:100)
F1
F2
F3
F4
F5
